# Supplementary material for: Fluid Flow Mechanical Stimulation-Assisted Cartridge Device for the Osteogenic Differentiation of Human Mesenchymal Stem Cells
Source: Micromachines (Basel). 2021 Aug 3;12(8):927. doi: 10.3390/mi12080927 (PMC8398302; doi:10.3390/mi12080927)
Supplement: Supplementary file 1 [file micromachines-12-00927-s001.zip › micromachines-1311910-supplementary.pdf]

Fluid flow mechanical stimulation-assisted cartridge device for the osteogenic differentiation of human mesenchymal stem cells

Ki-Taek Lim , Dinesh-K. Patel , Sayan-Deb Dutta and Keya Ganguly

Figure(s):

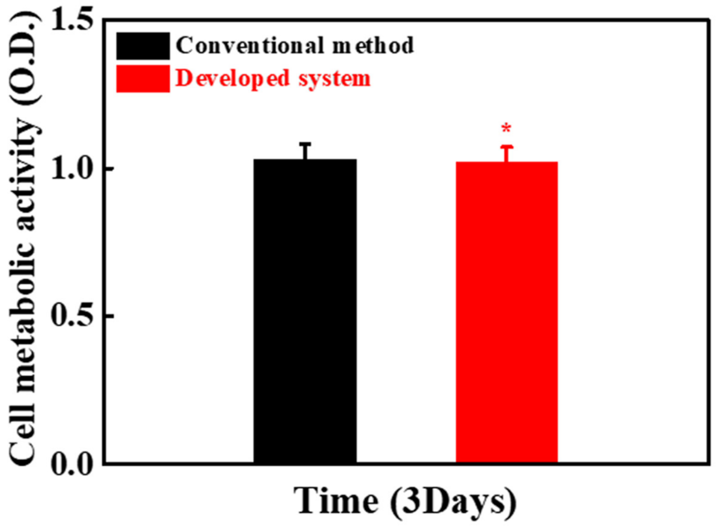

Figure S1. Comparative study of hMSCs viability between the conventional method (incubated at 5% CO_2 incubator), and the developed system (static condition) after 3 days.
